# A cross-sectional study on *Campylobacter fetus* subsp. *venerealis* prevalence and associated factors in Brazilian southern cattle farms

**DOI:** 10.1007/s42770-023-01119-7

**Published:** 2023-09-09

**Authors:** Franciele Maboni Siqueira, Gabriela Merker Breyer, Silvia De Carli, Cassiane E. Lopes, Maria Eduarda Dias, Maria Eduarda Rocha Jacques da Silva, Camila Moni, Larissa Caló Zitelli, Márcio Borsato, Rogers Gomes, Francisco Paulo Nunes Lopes, Rosane Collares Moraes, Milton Cattáneo, Ruben Sosa, Gilson Antonio Pessoa, Eduardo de Freitas Costa

**Affiliations:** 1 Laboratory of Veterinary Bacteriology, Department of Veterinary Clinical Pathology, Federal University of Rio Grande Do Sul, Porto Alegre, RS Brazil; 2 Postgraduate Program in Veterinary Sciences, Federal University of Rio Grande Do Sul, Porto Alegre, RS Brazil; 3 Laboratorios Microsules Uruguay S.A, Canelones, Uruguay; 4 Secretary of Agriculture, Livestock, Sustainable Production and Irrigation From Rio Grande Do Sul State, Porto Alegre, Brazil; 5 Embryolab–Animal Embryology Laboratory, Department of Large Animal Clinical (DCGA), Federal University of Santa Maria (UFSM), Santa Maria, RS Brazil; 6 Department of Epidemiology, Bio-Informatics and Animal Models, Wageningen Bioveterinary Research, Lelystad, The Netherlands

**Keywords:** Bovine genital campylobacteriosis, Venereal diseases, Survey, Extensive cattle farming, Cattle infertility

## Abstract

Cattle farming is a major livestock activity with economic relevance in Rio Grande do Sul (RS), Brazil. However, this activity is still considered of intermediate to low technological level, and in this region, there are few epidemiologic reports of *Campylobacter fetus* subsp. *venerealis* (Cfv), the causative agent of bovine genital campylobacteriosis (BGC). Thus, we designed a cross-sectional study to assess the prevalence and Cfv-associated factors in cattle farms in RS, Brazil. In total, 99 farms were randomly selected to participate in the survey. Preputial mucus samples from selected bulls were collected twice (within a 15-day interval) and subjected to Cfv molecular detection. A farm was considered positive when at least one sample was positive for Cfv. Our findings indicate that the farm-level Cfv prevalence in RS is 67.67%. On average, the chance of a farm using natural service to be Cfv-positive increased approximately twice compared to farms that do not use natural service. We also determined that Cfv routine tests reduce the chance of a farm being positive by 92%. Therefore, both Cfv detection tests and the reduction of natural services decrease the chance of a farm being positive for Cfv. Finally, we conclude that Cfv is widely spread in Southern Brazil cattle farms and it is urgent the implementation of control measures to reduce Cfv prevalence in the target population.

**Supplementary Information:**

The online version contains supplementary material available at 10.1007/s42770-023-01119-7.

## Introduction

Cattle farming is a major livestock activity in Rio Grande do Sul (RS), Brazil. In 2021, RS exported USD 308.5 million in beef to 90 countries [1]. Despite the economic importance, cattle farms in both RS and Brazil mainly adhere to an extensive production system, with an intermediate to low technological level, and few investments in biosecurity and disease control [2].

Bovine genital campylobacteriosis (BGC) is the main cause of reproductive issues in extensive cattle and low-tech livestock, placing important economic losses and restrictions on animals or animal products trade [3]. BGC is caused by the bacterium *Campylobacter fetus* subsp. *venerealis* (Cfv), which shows an important parasitic relationship, and a high adaptation to the bovine genital tract [4]. Bulls are asymptomatic Cfv-carriers in the herd, lodging Cfv in the preputial crypts and transmitting it to heifers/cows through coitus [5]. In females, most infected animals are stricken with a self-limiting infection, but some animals are affected by chronic or acute inflammatory disease that may result in reproductive losses. Brazil has no official BGC surveillance, and the available data is originated from convenience samples. Consequently, there is a lack of up-to-date information on the prevalence of positive farms and the factors associated with positive farms in Brazil, including RS state. Thus, we aimed to perform a cross-sectional study to assess the prevalence of Cfv in beef cattle farms in RS, Brazil, and describe the factors associated with Cfv infection in the target population.

## Materials and methods

### Study design and sample collection

RS is the southernmost state of Brazil, bordering Uruguay in the south, Argentina in the west and northwest, and the Brazilian state of Santa Catarina in the north. According to the official data from the Secretary of Agriculture, Livestock, Sustainable Production, and Irrigation (SEAPI-RS), the RS hosts ∼178,138 cattle farms, of which approximately 80% had up to 50 animals, 17% up to 500 animals, and 1.9% up to 10,490 animals [1]. The production system is extensive, with animals feeding natural pastures or annual grass. The target population comprised 13,054 commercial cattle farms with both more than 100 animals and holding bulls for reproduction purposes, which are unevenly distributed in the different RS regions, as follows: Center-occidental (14.87%), Center-oriental (3.43%), Metropolitan of Porto Alegre (5.9%), Northeast (5.6%), Northwest (8.57%), Southeast (17.85%), and Southwest (43.77%).

**Fig. 1:**
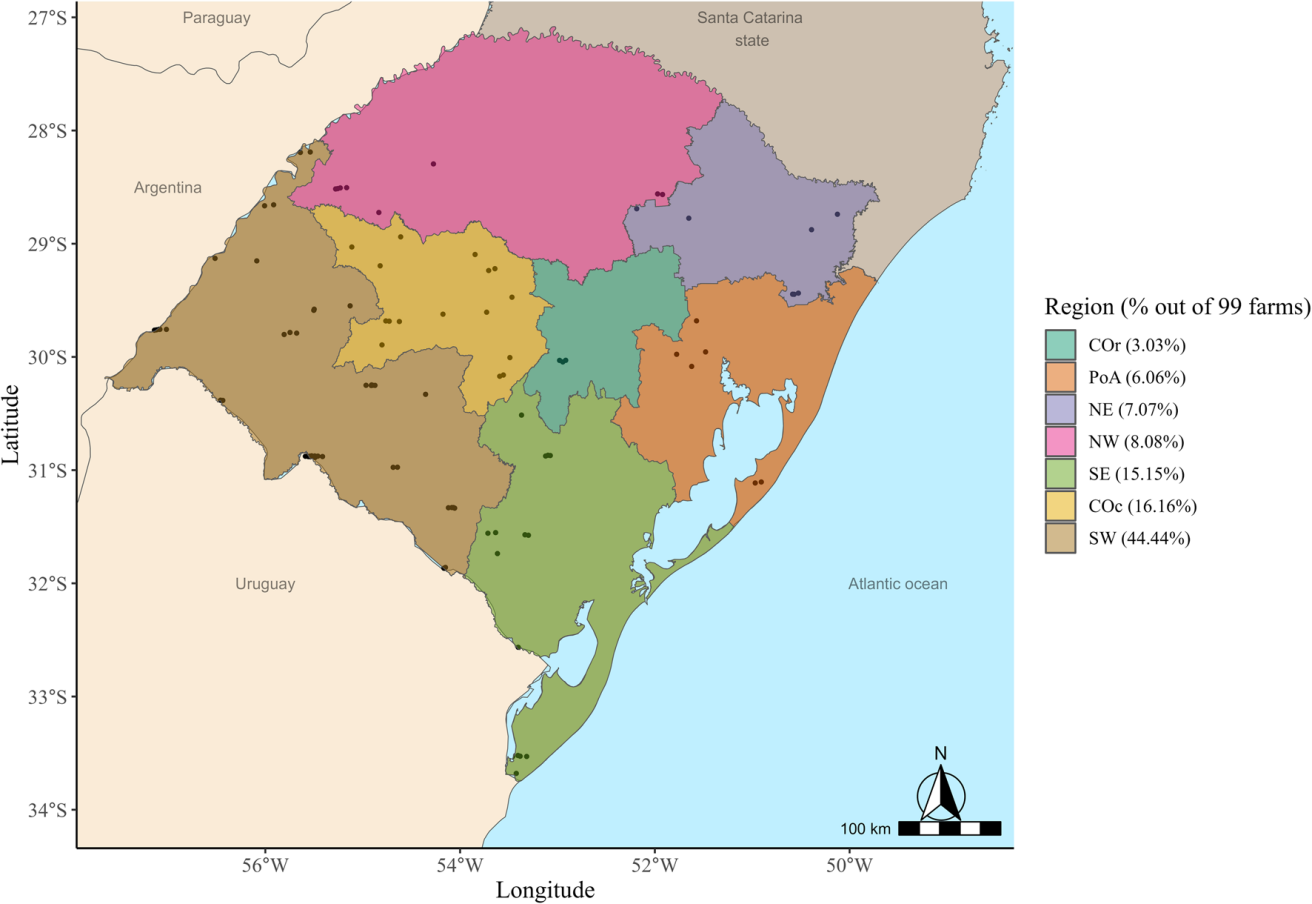
Geographic distribution of the studied population across the seven regions in Rio Grande do Sul State–Brazil*.* COc Center-occidental, COr Center-oriental, PoA Metropolitan of Porto Alegre, NE Northeast, NW Northwest, SE Southeast, and SW Southwest

A stratified two-level sample design was used. The primary unit was the herd, stratified along the seven RS regions and randomly selected (Fig. 1) from a list of farms available at the local veterinary services office. A minimum sample size of 97 farms was calculated to estimate a prevalence of 50% with a confidence level of 95% and precision of 10% [6]. The distribution of sampled farms per region is available in Fig. 1. Bulls were the secondary sample units drawn from each herd to account for 95% of the probability of detecting at least one positive bull in each herd, assuming 30% within-herd prevalence (average animal-level prevalence obtained from De Carli et al. [7], Ziech et al. [8], and Balzan et al. [9]), following a hypergeometrical process for a finite population. In each participating farm, available information about farm management was recorded (Table 1).

**Table 1:** Description of the variables obtained during the visit for sample collection in all 99 studied farms in Rio Grande do Sul–Brazil

Variable	Type	Description	Categories
Region	Categorical	Macro-region of Rio Grande do Sul state	Center-occidental, Center-oriental, Metropolitan of Porto Alegre, Northeast, Northwest, Southeast, and Southwest
Vaccine	Dichotomic	Use of vaccination against *Campylobacter fetus*	Yes or no
Type of farm	Dichotomic	Type of livestock system	Complete cycle or raising and restocking
Bull sale	Dichotomic	Frequent acquisition of new bulls	Yes or no
Natural service	Dichotomic	Use of bulls to breed females not pregnant after artificial insemination	Yes or no
Cfv* test	Dichotomic	Routine testing in the farm for Cfv detection	Yes or no

*Cfv Campylobacter fetus subsp. *venerealis*

Sample collection and molecular analysis were performed between July 2020 and December 2021. The procedures were approved by the Ethics Committee for Animal Experimentation from UFRGS n. 42052. In total, 99 farmers were actively contacted by phone being invited to participate in the project. With the farmer’s agreement and the confirmation of the squeeze chute to bulls’ restraint, the visits were scheduled, and the bulls were collected. Figure 1 shows the geographical location of each sampled farm from Rio Grande do Sul State–Brazil. For logistical reasons, within each farm, bulls were selected by convenience, ensuring sexual rest for at least seven days before collection. Two samples were obtained within a 15-day interval. Preputial mucus from bulls was collected by scraping [10, 11]. Mucus samples were placed in sterile microtubes and stored in a cool box with ice during transport to the laboratory. The following laboratory analysis was performed within 48 h after collection. All the samples were collected by two well-trained veterinary researchers to avoid bias.

### DNA isolation and molecular detection of Campylobacter fetus subsp. venerealis

Genomic DNA extraction from the collected samples was performed, as previously described [11, 12], using PureLink® Genomic DNA Mini Kit (Invitrogen) according to the manufacturer’s instructions. DNA integrity was evaluated by electrophoresis on agarose gel 0.8% stained with 40 × UniSafe Dye (Uniscience Corporation) and quantified by spectrophotometry. Internal control of the nucleic acid extraction from all samples was performed by 16S rDNA amplification using universal primers (27F: 5′-AGAGTTTGATCMTGGCTCAG-3′ and 1492R:5′-GGTTACCTTGTTACGACTT-3′).

Cfv molecular identification was performed by polymerase chain reactions (PCR) targeting ISC*fe1* (ISC1-F: 5′-GGTGGAGAGCGTAGATATAAATTAG-3′ and ISC1-R: 5′ CCATAAAGCCTAGCTGAAAAAACTG-3′) [13]. Each PCR reaction contained 0.5 U of GoTaq DNA Polymerase (Promega), 1 × buffer, 0.4 μM of each primer, 0.2 mM of dNTP, and 30 ng of DNA template. The PCR amplification conditions included initial denaturation at 95 °C for three min, followed by 35 cycles of 95 °C for 20 s, annealing at 54 °C for 20 s, and extension at 72 °C for 2 min, with a final extension of 72 °C for 10 min. Cfv ATCC 19438 was used as a positive control, while the negative control comprised reactions without DNA template. PCR products were evaluated in agarose gel electrophoresis and were sequenced by Sanger methodology to confirm Cfv detection. We considered the farm as Cfv-positive when at least one bull was Cfv-positive in at least one of the two samples obtained within the 15-day interval [3].

### Statistical analysis

A descriptive statistical analysis was used to explore the characteristics of the study population. To assess the factors associated with the presence or absence of Cfv in each farm, a generalized linear model [14] was applied using the logit link function and a “residual” Bernoulli distribution. The linear predictor of the model included fixed effects for the region, vaccine use, type of farm, bull’s sale, and natural service (Table 1). Artificial insemination was highly correlated with the use of natural services, and therefore, it was not included in the model to avoid multicollinearity issues. All variables were offered to the multivariable model using a backward selection procedure starting with the statured model and sequentially removing the largest *P*-value until only significant variables remained in the model (i.e., *P*-value ≤ 0.05). The variable number of bulls was assumed to represent the size of the herd and used as a confounder in the analysis. The generalized linear model was implemented in routine glm from the base library; fixed effects were tested with the classic Wald test, employing an approximation by the chi-square distribution. Multicollinearity was assessed by calculating the variance inflation factor (VIF) using two as the cut-off for multicollinearity. The Wald tests and VIF were assessed from the car library. All analyses were done in R [15].

## Results and discussion

**Fig. 2:**
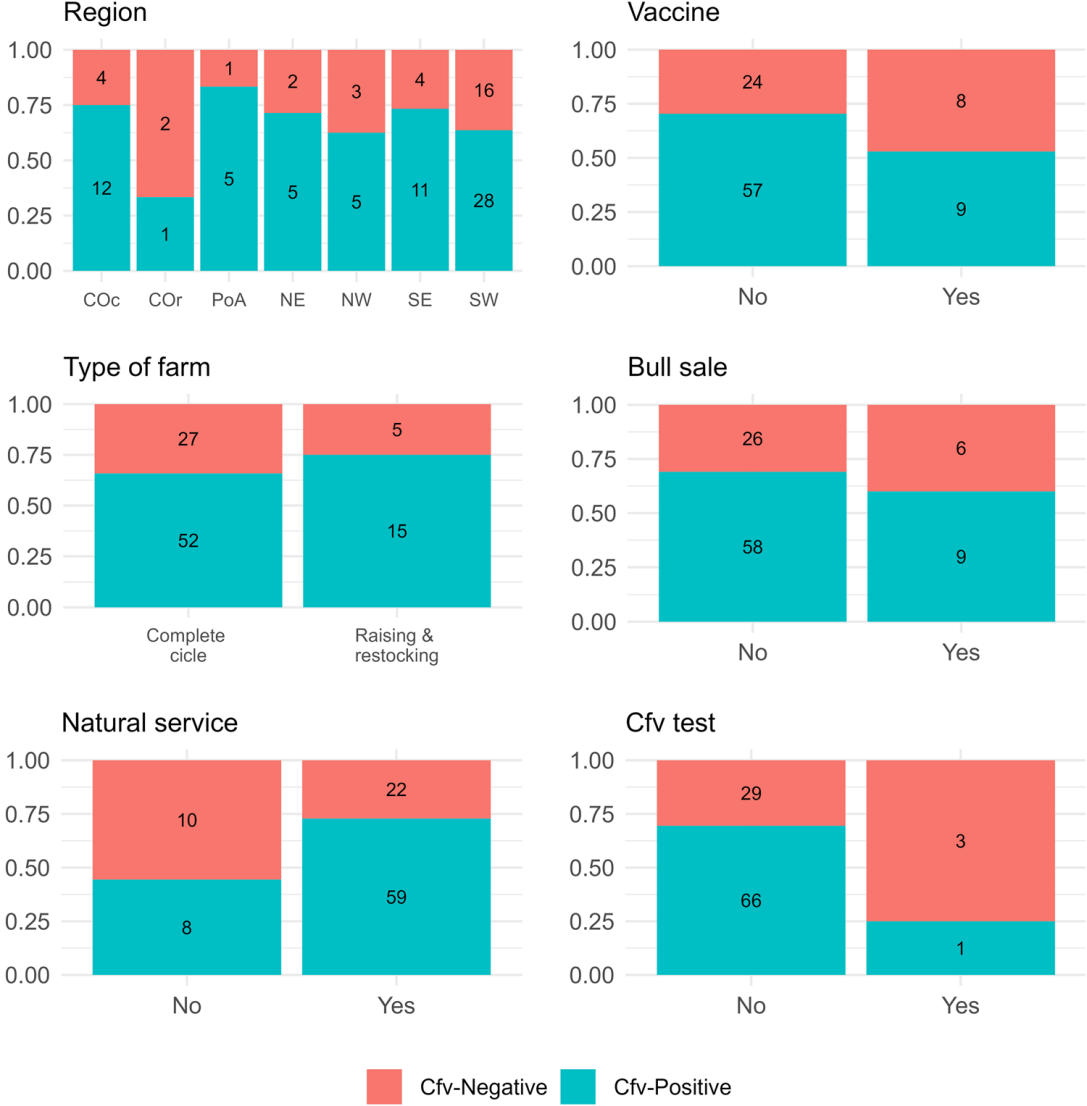
Distribution of the studied population characteristics and the relative frequency of *Campylobacter fetus* subsp. *venerealis* (Cfv) positive farms*.* COc Center-occidental, COr Center-oriental, PoA Metropolitan of Porto Alegre, NE Northeast, NW Northwest, SE Southeast, and SW Southwest

Among all 99 farms visited, 485 bulls were sampled, and the number of tested bulls per farm ranged from one to 20. According to the analyses, 29.3% of the sampled animals were Cfv-positive in at least one sample. Figure 2 shows the overall descriptive statistics for the management characteristics in the studied farms. The distribution of the Cfv-positive farms per region was evenly distributed, except for the Central-oriental region, where the frequency is lower (Fig. 2). Vaccine, type of farm, and bull sales had a balanced proportion of positive and negative farms. On the other hand, the frequency of positive farms is higher in farms using natural service after artificial insemination and in farms not testing for Cfv.

The overall farm-level prevalence of Cfv in RS is 67.67%, (58.1–76.35%), with 95% confidence interval (CI), showing that this agent is extensively present in the target population. Similarly, De Carli et al. [12] also reported a high within-herd prevalence (50%) in RS using the same molecular diagnosis technique and a convenience sample. Other studies in the same population, however, reported lower prevalence. Ziech et al. [16] observed 10.9% prevalence using laboratory-acquired samples, and Balzan et al. [17] reported an 8% animal-level prevalence from convenience samples between 2011 and 2018. Nevertheless, these authors used culture isolation for sample screening, which is known to have lower sensitivity than molecular identification given the fastidious growth of Cfv [18]; also, these studies did not use a proper study design for prevalence estimation.

After five steps, the final regression model had Akaike criteria information of 121.96, retaining two variables (Supplementary Table 1). On average, natural service increases the chance of a farm being Cfv-positive more than twice (i.e., exp (1.27)-1) compared with farms that do not use this reproductive management (Table 2). Accordingly, natural service for breeding has already been reported as a risk factor for Cfv [19]. This reproductive management is commonly used in RS when artificial insemination (AI) does not work or as a complementary practice after AI to ensure cows’ pregnancy. Consequently, natural service and AI are correlated, so we dropped the AI out of the model to avoid multicollinearity issues. Thus, we could hypothesize that, in the natural service system, there is continuous exposure of bulls to cows with reproductive failures helping to maintain the infection in the herd. Another bull-related aspect documented in the literature is the bull’s age being a risk factor for Cfv infection [20]. In this study, we did not have access to the reliable animals’ age because the farmers are not used to keeping a register of animals on the farm, but it should be considered in future studies.

**Table 2:** Summary of the final multivariable model to assess the prevalence and associated factors with *Campylobacter fetus* subsp. *venerealis* infection in bulls in Rio Grande do Sul-Brazil cattle farms

Variables*	Estimate (SE‡)	Odds ratio (95% CI §)	Prevalence	*P*-value
Intercept	− 0.4 (0.5)	-	-	-
Natural service				0.021
No	-	1	19.7%	
Yes	1.27 (0.55)	3.57 (1.21–10.8)	46.6%	
Cfv test†				0.023
No	-	1	62.1%	
Yes	− 2.53 (1.22)	0.08 (0.003–0.71)	11.5%	
Number of bulls	0.017 (0.016)	-	-	0.23

*The variance inflation factor (VIF) was lower than two for all variables in the final model

^†^*Cfv Campylobacter fetus* subsp. *venerealis*

^‡^*SE* standard error

^§^*CI* confidence interval

In addition, we observed that using Cfv routine tests reduces the chance of a farm being Cfv-positive by 92% (i.e., exp (-2.53)-1) on average. Although the number of bulls is not statistically significant, it was pushed in the model as a confounder (Table 2). In detail, in the studied population, only four farms perform routine tests for Cfv detection. However, it was enough to capture the protective effect of testing on the chance of positivity for Cfv*.* Moreover, bulls are long-term asymptomatic carriers, so it is impossible to identify Cfv-positive individuals in the herd beyond testing [21]. Further studies with more samples could be beneficial to reduce the uncertainty around the association between testing and the chance of Cfv detection in farms.

In Brazil, the low number of diagnostic laboratories for Cfv and the limited knowledge about the spread of this pathogen corroborate the lack of Cfv routine tests to control its introduction and maintenance in the herds. In the neighboring country Argentina, for instance, a regional Program for the Control and Eradication of BGC has taken place since 2006, keeping the infection controlled quite effectively and continuously following up the epidemiological status [22]. Therefore, investment in biosecurity measures for BGC control should be discussed and improved since Cfv testing could benefit the farmers by reducing Cfv exposure and prevalence in the herds.

Nevertheless, this study has some limitations regarding the Cfv-associated factors. For instance, the vaccine showed no association with Cfv presence, although it is supposed to protect herds by reducing Cfv infection rates. This may be because cross-sectional studies may have low power to detect association as sample calculation aims to estimate a proportion but not differences between variables. Another limitation common to observational studies is the fact that, despite all study design and sample size calculation, the samples cannot be totally selected randomly because either the farmers must to consent participation or because collecting the bulls is a hard task, demanding a deep involvement of the farms’ workers. However, such studies are the first step toward eliciting epidemiological evidence in a population [23], and further studies could use more robust designs toward risk estimation, e.g., prospective studies, calculating the sample size aiming the power to detect associations. In addition, we observed in this study the low number of farms using vaccines and routine Cfv testing, which is in accordance with the lack of biosecurity livestock sector in RS. Consequently, biosecurity variables were not implemented in this population or not recorded, making it difficult to use these data to determine factors associated with Cfv infection. However, we highlight that these limitations do not invalidate the findings observed. Cfv is widely spread in Southern Brazil cattle farms, and it is urgent to implement control measures to reduce Cfv prevalence in the target population. We conclude that, in this population, two important management practices are associated with Cfv prevalence. The routine testing in the farm for Cfv detection was observed to be effective in reducing the between-farm prevalence and using natural services increases the chance of a farm being positive for Cfv.

### Supplementary Information

Below is the link to the electronic supplementary material.

Supplementary file1 (DOCX 15 KB)

## Data Availability

The datasets generated during the current study are not publicly available to protect farmers' privacy and confidentiality, but relevant data is available from the corresponding author on reasonable request.
