# Supplementary material for: A cross-sectional study on Campylobacter fetus subsp. venerealis prevalence and associated factors in Brazilian southern cattle farms
Source: Braz J Microbiol. 2023 Sep 9;54(4):3291–7. doi: 10.1007/s42770-023-01119-7 (PMC10689683; doi:10.1007/s42770-023-01119-7)
Supplement: Supplementary file 1 — Supplementary file1 (DOCX 15 KB) [file 42770_2023_1119_MOESM1_ESM.docx]

**Supplementary Table 1** - Summary of the backward logistic regression model used to assess the variables associated with *Campylobacter fetus* subsp. *venerealis* in cattle farms in Rio Grande do Sul-Brazil.

| Step 1 **(saturated model)** | P value | **AIC=130.59** |
| --- | --- | --- |
| Region | 0.65 |  |
| Vaccine | 0.29 |  |
| Exploration | 0.71 |  |
| Bull sale | 0.35 |  |
| Natural service | 0.002 |  |
| Cfv test | 0.09 |  |
| N of bulls | 0.13 |  |
| Step 2 dropping **type of farm** |  | **AIC=128.7** |
| Region | 0.65 |  |
| Vaccine | 0.27 |  |
| Bull sale | 0.36 |  |
| Natural service | 0.0019 |  |
| Cfv test | 0.08 |  |
| N of bulls | 0.14 |  |
| Step 3 dropping **region** |  | **AIC=120.87** |
| Vaccine | 0.24 |  |
| Bull sale | 0.13 |  |
| Natural service | 0.007 |  |
| Cfv test | 0.022 |  |
| N of bulls | 0.14 |  |
| Step 4 dropping **vaccine** |  | **AIC=120.82** |
| Bull sale | 0.076 |  |
| Natural service | 0.009 |  |
| Cfv test | 0.011 |  |
| N of bulls | 0.13 |  |
| Step 5 dropping **bull sale** |  | **AIC=121.96** |
| Natural service | 0.021 |  |
| Cfv test | 0.023 |  |
| N of bulls | 0.25 |  |
